# Referral letters to pediatric rheumatology: referral content and impact on triage – an observational study

**DOI:** 10.1186/s12969-025-01150-y

**Published:** 2025-10-10

**Authors:** Alexandra Rydz, Fangfang Fu, Zhe Lu, Mark Drew, Dax Rumsey, Yan Yuan, Mercedes Chan

**Affiliations:** 1 Department of Pediatrics, University of Alberta, Edmonton, Canada; 2 Department of Mathematical and Statistical Science, Faculty of Science, University of Alberta, Edmonton, Canada; 3 School of Public Health, University of Alberta, Edmonton, Canada; 4 Department of Pediatrics, Division of Pediatric Rheumatology, University of British Columbia and BC Children’s Hospital, K4-118, 4480 Oak Street, Vancouver, BC V6H 3V4 Canada

**Keywords:** Referrals, Triage, Access to health care, Quality improvement

## Abstract

**Background:**

Delays in access to care in rheumatology are well documented. Many factors contribute to these delays and studies in adult populations indicate that incomplete referral letters may play a role. This study aims to describe the content of referral letters to pediatric rheumatology and to investigate the impact of incomplete letters on time to triage and accessing care.

**Findings:**

**Methods:**

We evaluated referrals to a tertiary care pediatric rheumatology centre for eight components of comprehensive referral letters. In addition, we compared time-to-triage and percentage of patients receiving rheumatic diagnoses between letters with sufficient content for immediate triage versus incomplete letters requiring further information. Logistic regression models identified factors associated with delayed triage.

**Results:**

Further information was requested for 67/447 (15%) referrals, resulting in median delay in time-to-triage of seven days. Delayed triage was associated with four factors: lack of musculoskeletal physical examination, referral from family physicians versus other specialty, missing information regarding management, and lack of rheumatic diagnosis of concern. Rheumatic diagnoses resulted from 42% of all referrals overall, specifically from 170/384 (44%) of immediately triaged referrals and 19/63 (30%) of referrals requiring further information. Rheumatic diagnoses resulted less commonly from family physician referrals.

**Conclusions:**

Missing important details in referrals to pediatric rheumatology contribute to delayed assessment. These findings can inform initiatives to educate physicians around relevant content of referral letters to facilitate timely access to care.

## Background

Delays in access to care in pediatric rheumatology (PR) are well documented [1, 2] and multifactorial [3]. Referral letters are one potential contributor to delayed access. Effective triage requires relevant medical history and physical examination, yet these details are often lacking [4–8]. Adult rheumatology studies suggest an association between incomplete referral letters and increased wait times [9, 10], but little is known about referral letters to PR. Identifying deficiencies in referrals can critically inform educational interventions for referring physicians to optimize patient outcomes. Our study describes the content of referral letters to PR at a tertiary care center and the impact of incomplete referral letters on time-to-triage for PR assessment.

## Methods

New referral letters to PR at a Canadian tertiary care centre were evaluated weekly by pediatric rheumatologists over a 22-month period. Letters are received via fax or the electronic health records system by a division administrator who prints all referrals and brings them to a weekly triage meeting (Monday mornings). Appointment dates are offered based on a time frame, e.g., within four weeks, that triaging doctors decide on as a group at the triage meeting. Patients are offered appointments with the first available physician with that time availability. However, final appointment times may depend on patient factors such as availability of transportation, funding for travel, or ability to get time off work for caregivers.

Triage decisions were first made by the physician group which is our usual practice, including requests for more information where required. Following this, letters were benchmarked against a referrals’ checklist from an existing regional health authority resource [11], adapted for PR practice by adding musculoskeletal (MSK) examination and suspected rheumatic diagnosis as checklist items. Letters were reviewed for eight components of a high-quality referral: suspected rheumatic diagnosis; patient symptoms; investigations; general physical examination; MSK examination; co-morbidities; current and past management; and medications. Basic patient demographics and referring physician specialty were also collected. Exclusion criteria were age > 17 years at time of referral; previously diagnosed rheumatic disease; and referral description that was clearly non-rheumatic in nature (and thus declined). We calculated a sample size of 450 referrals to have 80% power to detect risk factors for delayed triage with an odds ratio of two.

Date of referral receipt, triage decision, and resultant PR appointments were recorded. Time from referral receipt to PR appointment was calculated. Where incomplete referrals resulted in requesting additional information from referring physicians, the resultant delay in triage time was calculated. A multivariable logistic regression model identified factors contributing to delayed triage. All analyses were performed using R Statistical Software (v4.1.0) [12]. Final diagnoses were abstracted from medical records. Three pediatric rheumatologists reviewed all diagnoses independently, then convened to achieve consensus (2/3 agreement) on rheumatic versus non-rheumatic diagnoses.

## Results

**Fig. 1:**
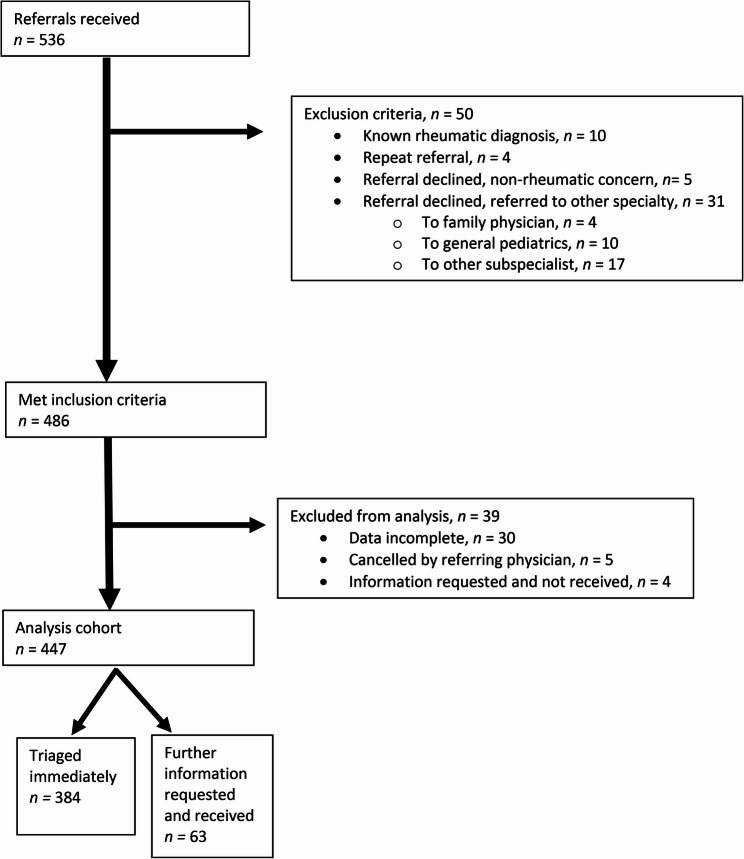
Flow diagram: referral screening and selection for analysis

Of 536 referrals received and evaluated, 89 were excluded (Fig. 1. Flow diagram: referral screening and selection for analysis). The remaining 447 referrals were from family doctors (45%); pediatric specialists and subspecialists (42%); and others (13%), including surgical subspecialists. Almost all referrals (94%) described patient symptoms; 55% and 48% included rheumatologic diagnosis of concern and MSK examination (Table 1. Patient, provider and referral letter characteristics, including final diagnoses stratified by time-to-triage).

**Table 1 Patient, provider and referral letter characteristics, including final diagnoses stratified by time-to-triage:** .

	No Delay, n=384	Delay, n=63	*P *value
Patient characteristics			
Age, mean (SD)	10.5 (4.6)	12.0 (4.1)	0.0153
Sex, n (% Male)	164 (42.7)	26 (41.3)	0.891
Referring physician specialities, n (%)			
Family medicine	155 (40.4)	47 (74.6)	<0.001
Pediatrics	175 (45.6)	13 (20.6)	
Other	54 (14.1)	3 (4.8)	
Number of letter components, mean (SD)	4.4 (1.7)	2.9 (1.4)	<0. 0001
Letter components, n (%)			
1. Rheumatic diagnosis of concern	226 (58.9)	18 (28.6)	<0.001
2. Symptoms	364 (94.8)	56 (88.9)	0.084
3. Lab investigations	266 (69.3)	41 (65.1)	0.558
4. Physical examination - musculoskeletal	203 (52.9)	10 (15.9)	<0.001
5. Physical examination – general	116 (30.2)	7 (11.1)	0.001
6. Current and past management	174 (45.3)	11 (17.5)	<0.001
7. Co-morbidities	161 (41.9)	19 (30.2)	0.096
8. Current medications	172 (44.8)	20 (31.8)	0.056
Rheumatic diagnosis, n (%)	170 (44.3)	19 (30.2)	0.039
Inflammatory arthritis, n (%)	90 (23)	12 (19)	0.333
Number of further referral letter components requested, mean (SD)	NA	2.97 (0.90)	NA
Further information requested, n (%)			
History	NA	57 (90.5)	NA
Diagnosis of concern		44 (69.8)	
Physical examination		57 (90.5)	
Investigations		29 (46.0)	

*SD* standard deviation, *NA* Not applicable

Overall, 188/447 referrals (42%) resulted in rheumatic diagnoses. Of those rheumatic diagnoses (*n* = 188), 55% were inflammatory arthritis, 14% were autoinflammatory disease, 7% were connective tissue disease, 4% were vasculitis and 20% were other rheumatic diagnoses. Rheumatic diagnoses resulted from 49% of pediatrician referrals and 37% of family physician referrals (*p* = 0.02). The median number of referral components for patient referrals resulting in a rheumatic diagnosis was 4 (IQR 3–6). Rheumatic diagnoses that most often had referral letters with ≥ 5 referral components were autoinflammatory diseases and other rheumatic diseases (42% each), juvenile idiopathic arthritis and vasculitis (38% each). 50% of all connective tissue diagnoses had referral letters with ≤ 4 referral components. Vasculitis diagnoses had referral letters with the fewest number of referral components, with 7/8 (88%) letters having ≤ 4 components.

Sixty-seven letters required further information from referring doctors prior to triage and consistently contained fewer referral components than letters triaged immediately (median 2.9 versus 4.4 components, respectively). Additional information was requested regarding pertinent history (91%), musculoskeletal examination (91%), rheumatologic diagnosis of concern (70%), and investigations (46%).

Additional information was received for 63/67 letters, resulting in a median delay in time-to-triage of 7 days (IQR 1–14). Information was received for four of these patients either the same or next day, facilitating immediate triage. Twelve of these 63 referrals (19%) resulted in a diagnosis of inflammatory arthritis. The median delay in time-to-triage for these 12 referrals was five days. The median time to first visit for patients diagnosed with inflammatory arthritis from the delayed triage group was 88 days (IQR 59–140), which was significantly longer compared to those diagnosed with inflammatory arthritis (*n* = 93) without delayed triage (median 46 days (IQR 26–75), *p* = 0.007).

Logistic regression analysis identified four independent factors contributing to delayed triage: a lack of MSK examination (adj. OR = 5.8), referral from family physician versus other specialty (adj. OR = 3.9), missing past or current management information (adj. OR 3.4), and a lack of rheumatic diagnosis of concern (adj. OR 2.4) (Table 2. Influence of missing elements from referral letters and of referring physician type on delayed triage). Patient age and sex, symptoms, general physical examination, and current medications did not affect time-to-triage, after accounting for the four factors listed above.

**Table 2 Influence of missing elements from referral letters and of referring physician type on delayed triage:** .

Missing Elements from Referral Letter	Adjusted Odds Ratio (95% CI)	P-value
Rheumatic diagnosis of concern	2.42 (1.28, 4.57)	0.007
Musculoskeletal physical examination	5.82 (2.79, 12.15)	<0.001
Current/past management	3.38 (1.64, 6.96)	0.001
Referring physician specialities, family physician versus other	3.88 (2.03, 7.43)	<0.001

## Discussion

### Quality of referral letters to pediatric rheumatology

Many referrals to PR lacked information necessary for effective triaging. Approximately 15% of referral letters in our study required further information, resembling the rate of incomplete referrals to pediatric specialists in other studies [13]. Although most referrals described patient histories, MSK examination was only included in half of referrals and was frequently requested for triage in addition to more history. These findings mirror referrals to adult rheumatology, where symptom duration and pattern of joint involvement are commonly omitted [4].

Complete referral letters are critical as they inform specialists’ decision-making for triage. In PR, inflammatory arthritis is a clinical diagnosis, based on history and physical examination. Several factors may contribute to the absence of pertinent histories and physical examinations. Educational gaps in rheumatic diseases may exist among the referring base of practitioners. In our study, referrals from family physicians were less likely to result in rheumatic diagnoses than those from pediatric providers and more often resulted in delayed triage. This likely reflects the decreased exposure family physicians have to pediatric rheumatic disease during their training compared to pediatric providers. Referring physicians who omit pertinent details on initial referrals may also lack knowledge of rheumatic diseases to provide requested or relevant details that convey a sense of urgency even after prompting, leading to delayed triage and later appointment dates. A lack of confidence in performing MSK assessments among clinicians in general [14, 15] is also recognized. Thus, referring physicians may opt to omit a physical examination rather than risk providing inaccurate examination findings. Finally, lack of details may simply reflect the reality of busy clinical practices.

### Impact of incomplete referral letters to pediatric rheumatology

Requests for information can delay triage. While this study found a median delay of only seven days, there is risk of more delayed triage if the referring physician cannot respond promptly. Some of our referrals were delayed much longer. Referring physicians may have had to call patients back to their clinics to complete a physical examination if it was requested and not originally performed. For some patients this may require organization of travel and childcare. Family physicians or general practitioners may also have been contacted for additional information when they were on leave or had a *locum tenens* covering who may not know the patient in question. Physicians may have also missed addressing our information requests due to the nature of busy practices and the burden of paperwork. While there is evidence that 36.4% of consultation requests to non–family physician specialists received no response within 5 to 7 weeks, leading to prolonged wait times and increased frustration for both patients and referring physicians [16], there is no literature examining responses to requests for additional information from specialists to referring physicians. Furthermore, requesting additional information poses a risk of loss to follow-up. In this study, four patients were not assessed because requested information was never submitted.

However, some letters that resulted in a rheumatic diagnosis had less than half of the desired referral components and this did not contribute to delay. Many of these referrals had indicated a specific diagnosis of concern or had relevant history to support the need for triage, e.g., patient with suspected granulomatosis with polyangiitis who had presented to Otolaryngology with a saddle nose deformity requesting further workup.

Delayed assessment in patients with inflammatory arthritis contributes to accrual of joint damage and disability. Unlike rheumatic diseases that present with acute symptoms expediting triage, e.g., vasculitis, inflammatory arthritis often presents insidiously. Children may go for weeks, months, or even years before accessing appropriate care. Despite the Arthritis Alliance of Canada’s recommendation that all inflammatory arthritis be identified and treated within four weeks of seeing a health professional, the median time from symptom onset to PR assessment is an estimated 268 days [2]. Like other rheumatology centres, inflammatory arthritis accounted for approximately half of the rheumatic diagnoses in this study [17].

Patients diagnosed with inflammatory arthritis following an initial incomplete referral letter had longer times to first visit than would be expected based on delayed triage alone. It is unclear why this would be given the median delay in triage time was only five days; however, it may be that the responses received were not worded to express urgent concern for inflammatory arthritis or need for medical attention, e.g., functional impairment. It is also well described that the most common presenting feature of inflammatory arthritis in childhood is joint swelling and not pain, the latter often alerting caregivers and physicians to seek care sooner [18]. Joint swelling, especially in younger children, or, for those unfamiliar with musculoskeletal examination, can be challenging to detect, and as such, may not be reported. Finally, our study did not examine whether dates of initial visits after triage were first available appointments, or if delays were incurred by patient preference, difficulties communicating with referring physicians, or other. Our pediatric rheumatology center is also a small one in a universal healthcare system. At the time of the study, we had three physicians whose workload totalled a 2.2 full-time equivalent. Clinic time could be limited and wait lists quite full; however, we always had a few spots available for urgent patients per week. Our catchment area is also quite large extending from our center to neighboring provinces and territories with transport often a challenge for patients living in more rural communities.

This is the first study to our knowledge to examine the content and impact of referral letters on time-to-triage in PR. While some factors associated with delayed triage, e.g., geographic location, cannot be modified, medical trainees and practitioners can be educated on essential components of referral letters and the triage process. At our institution, we actively involve trainees in triaging referral letters, with the goal of informing their future referral writing practices. Vora et al. [19] recently developed a PR triage algorithm to educate referring doctors on information required to facilitate referrals for effective triage. Advocating for pediatric rheumatology and pediatric musculoskeletal medicine to be mandatory in general pediatrics and generalist training and continuing medical education, and guidance on what complete referrals should entail during training may help close some of the educational gaps made apparent through incomplete referrals.

### Limitations

This study has strong internal validity, as it reflected local referral patterns, was adequately powered, and was conducted prospectively at weekly division triage meetings with a group of three physicians. Our findings may not be applicable to other centres due to inter-centre variability in triage processes and diagnoses considered appropriate for triage. Furthermore, our study did not control for occasions where staff rheumatologists discussed referrals via phone call or e-mail, which may have allowed for immediate triage. We also did not obtain details of treatments documented in referral letters which may have provided insight into how our referral base is treating patients with musculoskeletal/rheumatic complaints and help identify educational gaps relating to management. Finally, our study only evaluated times to in-person appointments, recognizing that with the rise of telehealth during the COVID-19 pandemic that some patients may have virtual consultations sooner now than if they had to be seen in-person only. The data obtained only reflects triage practices and cannot be extrapolated to further access to care.

## Conclusion

Incomplete referral letters result in delayed triage in PR. Improving knowledge of pediatric rheumatic disease and MSK examination skills may improve clinical assessments and referral content. Learning to write and triage referrals appropriately, including critical information that informs triage, as an entrustable professional activity will be an invaluable skill for graduating medical trainees. Because family physicians are more likely to refer a patient who ultimately does *not* receive a rheumatic diagnosis, educational initiatives may be most effective if prioritized for family medicine and generalist practitioners. A systematic checklist of required data to accompany referrals may also be helpful.

## Data Availability

The datasets used during the current study are available from the corresponding author on reasonable request.

## Abbreviations

MSK: Musculoskeletal
PR: Pediatric rheumatology
